# Drivers of facility deliveries in Africa and Asia: regional analyses using the demographic and health surveys

**DOI:** 10.1186/1742-4755-12-6

**Published:** 2015-01-16

**Authors:** Nadia Diamond-Smith, May Sudhinaraset

**Affiliations:** Global Health Group/Global Health Sciences, University of California, San Francisco, 550 16th Street, 3rd Floor, San Francisco, CA 94158 USA; Department of Epidemiology and Biostatistics and Global Health Group/Global Health Sciences, University of California, San Francisco, 550 16th Street, 3rd Floor, San Francisco, CA 94158 USA

**Keywords:** Facility delivery, Maternal health, Asia, Africa

## Abstract

**Background:**

In the past few decades many countries have worked to increase the number of women delivering in facilities, with the goal of improving maternal and neonatal health outcomes. The purpose of this study is to explore the current situation of facility deliveries in Africa and Asia to understand where and with whom women deliver. Furthermore, we aim to test potential drivers of facility delivery at the individual, household, and community-level.

**Methods:**

Demographic and Health Survey data collected since 2003 from 43 countries in Africa and Asia is explored to understand the patterns of where women are delivering. We look at patterns by region and wealth quintile and urban/rural status. We then run a series of multi-level models looking at relationships between individual, household and community-level factors and the odds of a woman delivering in a facility. We explore this for Asia and Africa separately. We also look at correlates of delivery with a trained provider, in a public facility, in a private facility, with a doctor and in a hospital.

**Results:**

The majority of women deliver in a facility and with a provider; however, about 20% of deliveries are still with no one or a friend/relative or alone. Rates of facility delivery are lower in Asia overall, and a greater proportion of deliveries take place in private facilities in Asia compared to Africa. Most of the individual level factors that have been found in past studies to be associated with delivering in a facility hold true for the multi-country-level analyses, and small differences exist between Asia and Africa. Women who deliver in private facilities differ from women who deliver in public facilities or at home.

**Conclusions:**

Most women in Africa and Asia are delivering in a facility, and drivers of facility delivery identified in smaller level or country specific studies hold true in multi-country national level data. More data and research is needed on other drivers, especially at the country-level and relating to the quality of care and maternal health complications.

## Introduction

Developing countries account for 99% of global maternal deaths, the majority in sub-Saharan Africa and Asia [1]. It is globally recognized that a challenge to achieving Millennium Development Goal (MDG) 5 – to reduce maternal mortality by 75% by 2015 - is the lack of access to skilled birth attendants at delivery. Increasing access to facilities is a recommended strategy to increase the number of women delivering with a skilled attendant because it leverages existing health system infrastructure and links women to referral systems, essential medical equipment and drugs in cases of complication [2].

Understanding why women decide to deliver in a facility is important for program and policy planning. A recent systematic review conducted by Moyer & Mustafa (2013) identified a number of drivers of facility deliveries in Sub-Saharan Africa, including maternal, social, facility, and macro-level factors [3]. The study found that maternal factors such as maternal education, parity/birth order, awareness of pregnancy risk factors, religion, and ethnicity were the most commonly studied drivers. However, the study found that less than two-thirds of the 65 studies included multi-variable analyses, and most studies focused on descriptive or bivariate analyses. There is significant variability in the sophistication of analyses across studies.

We conducted a review of the literature in Asian countries and found that, similar to Moyer and Mustafa’s findings from Sub-Saharan Africa, most studies focus on individual drivers. There is strong evidence that factors such as maternal education are associated with facility deliveries [4–11]. Other variables strongly associated with delivering in a facility include lower birth order [12–14], antenatal care visits [15–17], higher socioeconomic status [18–20], and living in an urban area [21–23].

Moreover, most studies focus on country-specific cases, rather than looking at drivers across regions, including smaller scale studies in Africa and Asia [24–29]. There is variability across geographic region and economic status on where women deliver [30]. An analysis of 48 Demographic Health Surveys (DHS) found that globally approximately half of births occurred at home [30]. Moreover, poor women in South Asia and Southeast Asia had higher levels of home deliveries compared to poor women in Sub-Saharan Africa (88.5%, 89.9%, and 77.7%, respectively). Given differences in rates of facility deliveries across regions, it is important to understand regional variations in why women choose to deliver at home or in a facility and to compare drivers and deterrents of delivering in a facility across regions.

This study builds upon existing literature and takes advantage of nationally representative data using the DHS to assess drivers of facility deliveries across multiple countries and regions. To our knowledge, an analysis of drivers of facility deliveries across Asia and Africa has not been undertaken with this dataset. The DHS allows us to go beyond individual factors and also examine, family, community, and facility-level factors associated with facility deliveries using more advanced multi-level modeling. A benefit of using this rich dataset is the common definition of facility delivery and providers across countries, and the ability to examine a multitude of individual and community-level factors across different contexts using more complex analytical tools. Whereas previous studies have looked at a single country, or a small handful of countries, and focused on a limited number of potential drivers, this study compiles data from over 40 DHSs and combines drivers of facility delivery at the individual, household and community level. Hence, we are able to test if the findings from a sub-set of countries are applicable across Asia and Africa, and if certain relationships hold after controlling for other potentially confounding drivers of facility delivery. Additionally, this study examines where deliveries are occurring across Africa and Asia, and highlights the types of facilities and providers in each region. The objective of the study was to describe the current landscape of facility deliveries, and to identify drivers of facility deliveries in Africa and Asia.

## Methods

### Data

We used data from 43 Demographic and Health Surveys (DHS) collected in the last 10 years (since 2003) to look at the current status of facility deliveries and related outcomes (Table 1). All countries in Africa or Asia, which had a DHS in the last 10 years, were included, and in countries with more than one DHS, we used only the most recent survey. DHS data is publically available and was downloaded from the MeasureDHS website (http://www.measuredhs.com). Data on the most recent birth was included, thereby limiting the analysis to one birth per woman, and only births occurring since 1993 are included in the analysis (the unit of analysis was an individual woman). Regions are classified based on the UN standards for sub-regions.

**Table 1:** **Demographic and health survey datasets used in this analysis**

	Year	Number of women	Region
**Angola**	2007	1,758	Southern Africa
**Bangladesh**	2011	14,895	Southern Asia
**Benin**	2006	13,251	Western Africa
**Burkina Faso**	2010	13,099	Western Africa
**Burundi**	2011	5,893	Eastern Africa
**Cambodia**	2010	11,345	Southeast Asia
**Cameroon**	2011	10,669	Middle Africa
**Chad**	2004	4,259	Middle Africa
**Congo**	2005	4,722	Middle Africa
**Cote D'Ivoire**	2005	1,920	Western Africa
**DRC**	2007	6,870	Middle Africa
**Egypt**	2005	14,971	Northern Africa
**Ethiopia**	2011	10,674	Eastern Africa
**Gabon**	2012	6,105	Middle Africa
**Ghana**	2008	3,136	Western Africa
**Guinea**	2005	5,853	Western Africa
**India**	2006	65,794	Southern Asia
**Indonesia**	2007	6,852	Southeast Asia
**Kenya**	2008	5,810	Eastern Africa
**Lesotho**	2009	4,880	Southern Africa
**Liberia**	2007	5,397	Western Africa
**Madagascar**	2008	12,333	Eastern Africa
**Malawi**	2010	17,656	Eastern Africa
**Maldives**	2009	5,760	Southern Asia
**Mali**	2006	11,123	Western Africa
**Morocco**	2004	7,135	Northern Africa
**Mozambique**	2011	10,270	Eastern Africa
**Namibia**	2006	6,152	Southern Africa
**Nepal**	2011	8,239	Southern Asia
**Niger**	2006	6,997	Western Africa
**Nigeria**	2008	22,997	Western Africa
**Pakistan**	2007	8,177	Southern Asia
**Philippines**	2008	7,951	Southeast Asia
**Rwanda**	2011	8,382	Eastern Africa
**Sao Tome**	2008	1,943	Middle Africa
**Senegal**	2010	10,449	Western Africa
**Sierra Leone**	2008	5,676	Western Africa
**Swaziland**	2007	3,175	Southern Africa
**Tanzania**	2010	7,101	Eastern Africa
**Timor-Leste**	2010	7,813	Southeast Asia
**Uganda**	2011	6,291	Eastern Africa
**Zambia**	2007	5,180	Southern Africa
**Zimbabwe**	2010	6,521	Eastern Africa
**TOTAL**		405,474	

We assessed the prevalence of facility deliveries, based on the reported place of last delivery. Facility delivery is defined as any place other than delivering at home, at someone else’s home, or en route to a facility. Public facilities are coded as any type of government facility (hospital, clinic, dispensary, etc.), and private facilities include any type of private institution (hospital, clinic, dispensary, etc.), maternity home, NGO or religious facility.

The DHS also asked women who attended their delivery. There are a large number of missing responses for this indictor, with about 29% of women not answering this question (these women were dropped from the analysis). The indicator of “delivery with a doctor or nurse” includes respondents who answered that they delivered with a doctor, nurse, nurse mid-wife, or auxiliary nurse mid-wife. “Traditional provider” includes traditional healers, traditional birth attendants, and homeopathic providers. “No provider” includes respondents who said no one, a friend, or a relative attended the birth. “Other health provider” includes anyone who was not in the previous three categories.

## Methods

We conducted a series of analyses using Stata 12MP. Demographic, household, and community drivers were modeled using logistic regressions on the outcome of facility- deliveries. Covariates were chosen based on the recently published systematic review of drivers of facility deliveries in Africa (Moyer & Mustafa, 2013). The maternal, social and antenatal care-related factors that they identified were used to select variables from the DHS to include in our analysis; however, facility-level factors identified in the review were not included because these questions were not asked in the DHS (Table 2).

**Table 2:** **Factors identified in Moyer and Mustafa (2012) paper and whether they are included in this analysis** [3]

Factors Listed in Moyer and Mustafa, 2012	Included in this analysis?
**Maternal Factors**	
Maternal Age	Yes
Maternal Education	Yes, years of education
Religion	No
Ethnicity	No
Region/province of residence	Yes, clustered by stratum
Urban/rural	Yes
Wealth/SES	Yes, wealth index
Maternal Employment	Yes, employed/not
Health insurance coverage	No, only collected in sub-set of countries
Parity/birth order	Yes, parity
Martial Status	Yes
Polygamous union	No, only applicable to a subset of countries
Empowerment/autonomy	Yes, score of acceptability of wife beating
Attitude towards importance of facility delivery/perceived need	No, not available
Attitude towards skills of doctor vs. TBA	No, not available
Embarrassment/fear of being shamed	No, not available
Discussion with male partner on place of delivery	No, not available
Knowledge of pregnancy risk factors	No, high levels of missing data
Completion of birth plan	No, not available
Concept of abnormal vs. normal pregnancy	No, not available
Having means of transport to facility/vouchers	No, not available
Quality of previous delivery	No, not available
Location of previous delivery	No, because not all women had a previous delivery
Pregnancy wantedness	Yes, desired pregnancy/not
Birth complications	No, not available
Use of herbal drugs	No, not available
Desire to appear modern	No, not available
Fear of episiotomy	No, not available
Precipitate Labor	No, not available
Use of maternity waiting homes	No, not available
**Social Factors**	
Non-male household head	Yes
Husband’s occupation	Yes
Husband/partner education	Yes
Small family norm (community level)	No, not available
Stigma/gossip/on lookers	No, not available
Living in socially disadvantaged neighborhood	No, not available
Permission from husband, TBA, mother, mother-in-law	No, not available
Social influence of others	Yes, stratum level acceptability of wife beating score
Village level: % of village who agree facility delivery is important	No, high levels of missing data
Village level: % of village who rated local facility as “excellent”	No, not available
Village level: % of village who attended 4+ ANC	Yes
Village level: % of village who agreed doctors and nurses have good skills	No, not available
Village level: % of village who agree TBAs have good skills	No, not available
Community perception of access to nearest facility	No, not available
Traditional views on delivery and motherhood	No, not available
**Antenatal care (ANC) factors**	
Attended ANC	Yes, any ANC
Timing of firs ANC visit (early)	No, not available
Number of ANC	Yes, 4+ ANC
Saw doctor at ANC	Yes
Quality of ANC	No, not available
Advised to deliver in a facility during ANC	No, not available

Multi-level analyses were used to explore correlates of facility deliveries and maternal, household, and community-level factors. The statistical model assumes that individuals’ health outcomes are partly dependent on the households and communities within which they live. This dependency is accounted for by separating individual from household and community level variation. Multi-level statistical techniques are described elsewhere in depth and provide a robust framework for disentangling different level effects on health outcomes [31]. None of the variables included in the model were strongly co-linear.

In the first set of models, the main correlate of interest is an individual’s probability of delivering in any facility (public or private; hospital, clinic, dispensary, etc.) compared to delivering at home. In the second set of models, the main correlate of interest is an individual’s probability of delivering with a provider (doctor, nurse, midwife). Demographic, household and community drivers are modeled separately, and then combined. All models are clustered at the stratum level, and weighted using information on the primary sampling unit and stratum. These models are then run separately for Asia and Africa. Finally, we re-run the global models on a series of more specific outcomes: delivery at a hospital compared to another facility or home; delivery in a public facility compared to another facility or home; delivery in a private facility compared to another facility or home; and delivery with a doctor specifically, compared to other health professional, no one, friend/relative or traditional provider.

## Results

Below we describe the pattern of where women deliver by region, and by wealth quintile and urban/rural status. We then describe the statistical analyses testing various drivers of facility delivery, and other outcomes including delivery with any trained provider, delivery at a hospital, in a public facility, in a private facility and with a doctor.

### Country-level descriptive analyses: place of delivery and provider type by region

**Figure 1:**
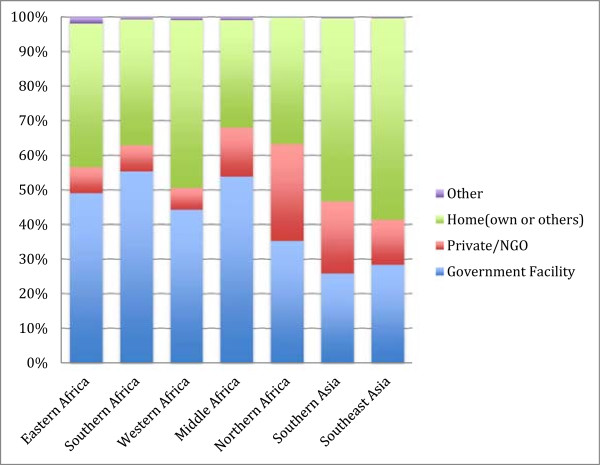
**Place of delivery of most recent birth, by region.**

The majority (53.4%) of women in Africa deliver in a facility. The rates are lower in Southern Asia (roughly 45%) and Southeast Asia (just over 40%) (Figure 1). The majority of deliveries that occur in a heath facility occur in government facilities in Africa, however, place of delivery is more evenly distributed between public and private facilities in Northern Africa and Southern Asia (and to some extent Southeast Asia).

**Figure 2:**
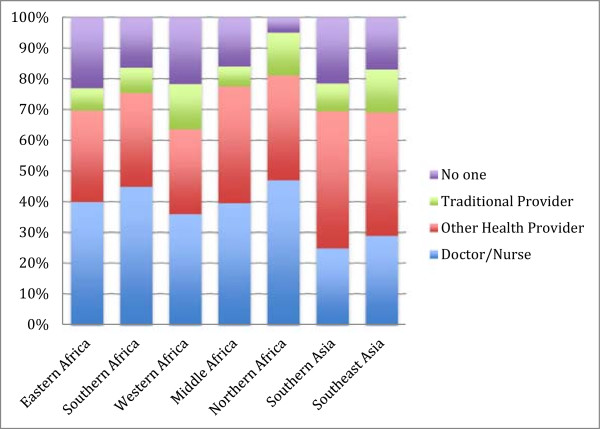
**Type of provider at most recent delivery, by region.**

In Africa and Asia, over 65% of deliveries are attended by some type of health provider (doctor, nurse, or other trained health provider) (Figure 2). In Africa, about 40% of deliveries are with a doctor or a nurse (with the exception of Western Africa, which is just above 35%). Attended deliveries are lower in Asia: between 25-30% of all births. Traditional providers attend between 5-15% of deliveries, with higher proportions in Northern and Western Africa and Southeast Asia. In all regions except North Africa between 15-20% of deliveries are reported as having no one, a friend or a relative present.

**Figure 3:**
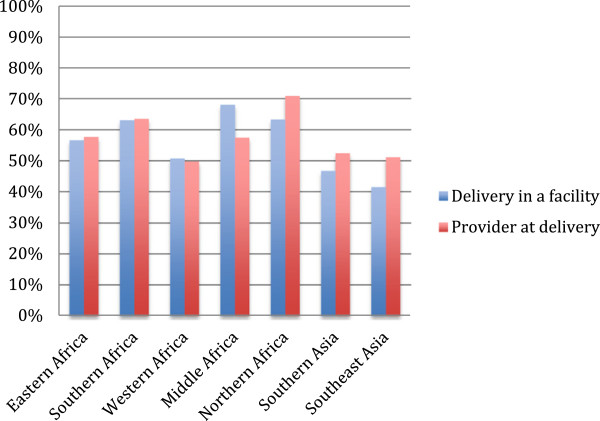
**Percent of deliveries in a facility and with a provider, by region.**

In most countries, delivering at a facility means that women are delivering with a trained provider (Figure 3). Middle Africa is an exception to this, where there is a substantial percent (>5%) of women who say they are delivering in a facility but are not reporting delivering with a health professional of some type. In Northern Africa and Southern and Southeast Asia there is gap between deliveries in a facility and delivery with a trained provider, with a substantial proportion of women (>5%) delivering with a trained provider outside of a facility setting.

### Inequalities: urban rural and wealth quintile differences in place of delivery by region

**Figure 4:**
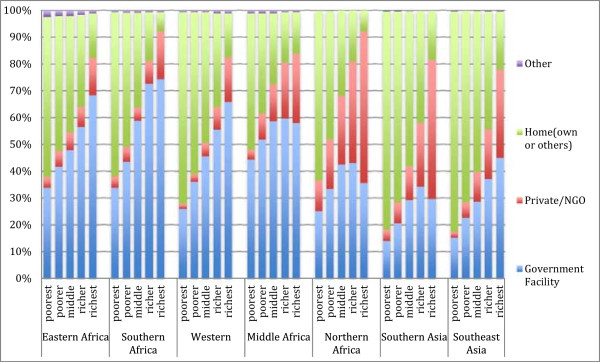
**Place of delivery by wealth quintile and region.**

**Figure 5:**
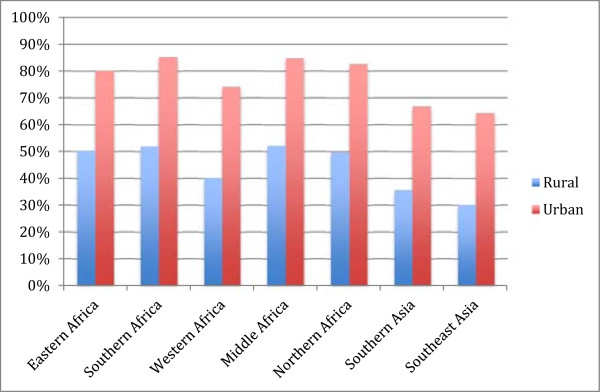
**Percent of deliveries in a facility in rural and urban populations, by region.**

As can be seen in Figure 4, poorer women, those in the lowest wealth quintile, are much less likely to deliver in a facility than richer women in all countries. Richer women are also much more likely to deliver in private facilities than poorer women. Inequalities in facility deliveries are greatest in Southern and Southeast Asia (with over 65% difference in facility delivery rates between the lowest and highest wealth quintiles). Middle Africa is the most equitable region, although inequalities still exist. Women who live in an urban area are much more likely to deliver in a facility than women living in rural areas (Figure 5). Again, the inequalities are greatest in Asia, but in all regions at least 30% more urban women deliver in a facility than rural women.

### Multivariate models: drivers of facility deliveries: individual, household, and community level factors

#### Descriptive characteristics

Over 400,000 women were included in the analysis which combined all 43 of the DHS’s (Table 1). O f those who answered the question about where they delivered, which includes 70% of the sample, about 54% of women delivered in a health facility (Table 3). Similarly, a mean of 55% of women said they delivered with a provider (of the roughly 70% of the sample that answered the question). Women were, on average, 31 years old and had 4.5 years of education. Over 80% of women in the sample were married and they had, on average, had given birth to 3.7 children. Almost 60% of women were employed and 73% stated that their last pregnancy was wanted at the time that they had it (however, this response was missing for 32% of respondents).

**Table 3:** **Descriptive of variables in the analysis**

Variable	Mean/Percent	Which countries missing entirely	Missing, N (%)
**Deliver in a facility**	53.5%		125, 462 (30.9%)
**Deliver with a provider**	54.9%		125, 410 (30.9%)
**Woman’s age**	31.4		0
**Woman’s education**	4.5 years		277 (0.07%)
**Total number of children ever born**	3.7		0
**Married**	80.3%	Angola	1,761 (0.4%)
**Woman employed**	58.6%	Angola, Bangladesh	22,181 (5.5%)
**Wanted last pregnancy at that time**	73.3%	Angola	128,499 (31.7%)
**Urban**	34.1%		0
**Wealth Index (quintiles)**	3.1		0
**Non-male household head**	19.3%		0
**Husband’s age**	38.7	Angola, Cote D’Ivoire,	53,819 (13.3%)
**Husband’s education**	5.7 years	Angola, Cote D’Ivoire, Maldives	33,245 (8.2%)
**Husband employed**	94.9%	Angola, Cote D’Ivoire, Bangladesh	33,245 (8.2%)
**Any ANC**	83.1%	Angola	133,117 (32.8%)
**More than 4 ANC**	49.4%	Angola	133,117 (32.8%)
**Saw doctor at ANC**	21.6%		126,321 (31.2%)
**Mean score of wife beating acceptability**	1.4	Angola, Chad, Congo, Cote D’Ivoire, Pakistan	21,684 (5.4%)
**Stratum level % of women with 4+ ANC**	36.9%		0
**Mean score of wife beating acceptability per stratum**	1.4	Angola, Chad, Congo, Cote D’Ivoire, Pakistan	21,684 (5.4%)

Thirty-four percent of the sample lived in urban areas, and the average wealth quintile was 3 (on a score from 1 to 5, poorest to richest) (Table 3). Nineteen percent of women lived in households with a non-male household head. Husbands/partners were, on average 38.7 years old (13.3% missing), had an average of 5.7 years of education, and 94.9% were employed in some type of occupation (8.2% missing).

Variables related to ANC visits had just over 30% of respondents missing (Table 3). Of those that answered these questions, 83% had at least one ANC visit, and 49% had four or more ANC visits. About 22% percent of the women reported that they saw a doctor at their ANC visit. The mean acceptability of wife beating score at the individual and community level was 1.4 (on a scale from 1 to 5, with a higher number meaning greater acceptability of wife beating under various circumstances). At a community level, an average of 36.9% of women in each community had had four or more ANC visits.

#### Multivariate model outcomes

Being older, having more years of education, having fewer children, not being married, the most recent baby being born more recently, not desiring the most recent pregnancy, having any ANC visits or 4 or more ANC visits, and having a lower score for community acceptability of wife beating were all significantly and robustly associated with increased odds of delivering in a facility (Table 4). Not being employed was significantly associated with increased odds of delivering in a facility in the Model with only demographic variables (Model 1.2), but not in the full model. Seeing a doctor or nurse was significantly associated with delivering in a facility in the full model, but not Model 1.2 with only demographic variables.

**Table 4:** **Model 1: probability of delivering in a health facility**

Odds of delivering in a health facility	(1): Full	(2): Demo-graphics	(3): House-hold	(4): Community
**Woman’s Age**	1.026***	1.043***		
**Woman’s Years Education**	1.041***	1.102***		
**Total Children Ever Born**	0.866***	0.848***		
**Married**	0.761***	0.724***		
**Woman Being Employed**	1.008	0.906**		
**Wanted Last Pregnancy Then**	0.864***	0.820***		
**Year of Most Recent Birth**	1.092***	1.075***		
**Four plus ANC**	1.491***	1.927***		
**Any ANC**	4.854***	6.164***		
**Saw Doctor at ANC**	1.136**	1.111		
**Acceptability of Wife Beating Score**	0.974***	0.961***		
**Urban**	1.638***		1.941***	
**Wealth Index**	1.385***		1.487***	
**Non Male Head**	1.109***		1.318***	
**Husband’s Age**	1.008***		0.997	
**Husband’s Years Education**	1.005		1.069***	
**Husband Being Employed**	0.698***		0.614***	
**Percent of stratum with 4 plus ANC**	8.716***			153.7***
**Mean Stratum level Acceptability of Wife Beating Score**	0.997			0.764***
**Country**	0.967***	0.970***	0.976***	0.979***
**Constant**	0***	0***	0.586**	0.495***
**Observations**	213,255	245,891	232,620	264,240

***p < 0.01, **p < 0.05, *p < 0.1.

In terms of household level factors, living in an urban (compared to rural) area, having a higher score on the wealth index, having a non-male household head, and having a husband who is not employed were significantly and robustly associated with increased odds of delivering in a facility (p < 0.01). Having a husband with more education was significantly associated with increased odds of delivering in a facility when only household factors were included in the model (Model 1.3), but it was no longer significant when other demographic factors were included. When only household factors were included (Model 1.3), husband’s age was not significantly associated with delivering in a facility, however, in the full model having an older husband was significantly associated with delivering in a facility. When only community level factors were included in the model (Model 1.4), a higher percent of the stratum who had 4 or more ANC visits was associated with an extremely high odds of delivering in a facility (OR = 153.7, p < 0.01), and a higher mean stratum level acceptability of wife beating was associated with lower odds of delivering in a facility. In the full model (Model 1.1) a higher percent of the stratum that had 4 or more ANC visits was associated with higher odds of delivering in a facility, and this value was of a reasonable magnitude (OR = 8.7, p < 0.01). The mean stratum acceptability of wife beating was not associated in the full model. Country of data collection was significantly associated in all models.

#### Delivery with provider

In Model 2 we explored correlates of delivering with a provider. These were very similar to the relationships found in Model 1 (delivering in a facility). Being older, having more years of education, having fewer children, not being married, the most recent baby being born more recently, not desiring the most recent pregnancy at this time, having any or 4 or more ANC visits, seeing a doctor for ANC, and having a lower acceptability of wife beating score were all significantly and robustly associated with increased odds of delivering with a provider (Table 5). Again, women’s employment status was only associated in Model 2.2, but not in the full model (Model 2.1).

**Table 5:** **Model 2: probability of delivering with a provider**

Odds of delivering with a health provider	(1): Full	(2): Demo-graphics	(3): House-hold	(4): Community
**Woman’s Age**	1.039***	1.050***		
**Woman’s Years Education**	1.051***	1.118***		
**Total Children Ever Born**	0.848***	0.830***		
**Married**	0.757***	0.744***		
**Woman Being Employed**	0.985	0.898***		
**Wanted Last Pregnancy Then**	0.881***	0.830***		
**Year of Most Recent Birth**	1.073***	1.062***		
**Four plus ANC**	1.514***	1.921***		
**Any ANC**	3.860***	5.032***		
**Saw Doctor at ANC**	1.433***	1.413***		
**Acceptability of Wife Beating Score**	0.977***	0.942***		
**Urban**	1.576***		1.832***	
**Wealth Index**	1.371***		1.497***	
**Non Male Head**	1.092***		1.314***	
**Husband’s Age**	1.003*		0.994***	
**Husband’s Years Education**	1.016***		1.083***	
**Husband Being Employed**	0.769***		0.728**	
**Percent of stratum with 4 plus ANC**	7.093***			136.8***
**Mean Stratum level Acceptability of Wife Beating Score**	0.912**			0.708***
**Country**	0.968***	0.971***	0.979***	0.979***
**Constant**	0***	0***	0.538**	0.635***
**Observations**	213,204	245,839	232,567	264,189

***p < 0.01, **p < 0.05, *p < 0.1.

In terms of household level factors, living in a urban (compared to rural) area, having a higher score on the wealth index, having a non-male household head, having a husband who is not employed and a husband with more education were significantly and robustly associated with increased odds of delivering with a provider (p < 0.01). When only household factors were included (Model 2.3), a younger husband was significantly associated with delivering with a provider, however, in the full model husband’s age was only marginally associated, and in the reverse direction. When only community level factors were included in the model (Model 2.4) and in the full model (Model 2.1), a higher percent of the stratum who had 4 or more ANC visits was again associated with an extremely high odds of delivering in a facility and a higher mean stratum level acceptability of wife beating was associated with a lower odds of delivering with a provider. Country of data collection was significant in all models.

#### Drivers by region: Africa and Asia

##### Africa

When only the countries in Africa are included in the model, being older, having more education, having fewer children, being married, not being employed, not desiring the most recent pregnancy at that time, having any or 4 or more ANC visits and a lower acceptability of wife beating were all significantly and robustly associated with increased odds of delivering in a facility (p < 0.01) (Table 6). The most recent birth being more recent was not significantly associated with facility delivery (only marginally in the full model, 3a.1). Living in an urban area, having a higher wealth quintile, the husband having more years of education, and the husband not being employed were significantly and robustly associated with delivering in a facility (p < 0.01). When only household factors were included (Model 3a.3) having a non-male household head, and the husband being younger were significantly associated, but these dropped out when other factors where included in the model (Model 3a.1). When only community level factors were included and when all factors were included, community level percent of women with 4 or more ANC visits was correlated with delivering in a facility. However, a lower mean-stratum level acceptability of wife beating was only significantly associated with increased odds of delivering in a facility in Model 3a.4. Country of data collection was significant in all models.

**Table 6:** **Model 3a: probability of delivering at a facility: Africa**

Odds of delivering in a health facility	(1): Full	(2): Demo-graphics	(3): House-hold	(4): Community
**Woman’s Age**	1.038***	1.042***		
**Woman’s Years Education**	1.061***	1.128***		
**Total Children Ever Born**	0.856***	0.839***		
**Married**	0.872**	0.876***		
**Woman Being Employed**	0.876***	0.822***		
**Wanted Last Pregnancy Then**	0.839***	0.797***		
**Year of Most Recent Birth**	1.034*	1.018		
**Four plus ANC**	1.328***	1.611***		
**Any ANC**	6.665***	8.398***		
**Saw Doctor at ANC**	0.837***	0.879**		
**Acceptability of Wife Beating Score**	0.975***	0.940***		
**Urban**	1.812***		2.147***	
**Wealth Index**	1.289***		1.398***	
**Non Male Head**	0.996		1.226***	
**Husband’s Age**	0.998		0.990***	
**Husband’s Years Education**	1.013**		1.090***	
**Husband Being Employed**	0.796***		0.708***	
**Percent of stratum with 4 plus ANC**	6.823***			97.26***
**Mean Stratum level Acceptability of Wife Beating Score**	0.924*			0.730***
**Country**	0.971***	0.972***	0.976***	0.982***
**Constant**	0**	0	0.881	0.652
**Observations**	154,174	181,630	166,970	191,237

***p < 0.01, **p < 0.05, *p < 0.1.

##### Asia

When only the southeast and southern Asian countries were included in the model, being older, having more education, fewer children ever born, the most recent baby being born more recently, not being employed, having any or 4 plus ANC visits and seeing a doctor for ANC were significantly and robustly associated with increased odds of delivering in a facility (Table 7). Marital status, whether the last pregnancy was desired at that time, and acceptability of wife beating were not significantly associated with the odds of facility delivery. Living in an urban area, having a higher wealth quintile, and a non-male household head were significantly and robustly associated with increased odds of delivering in a facility. However, the factors associated with the husband’s education were only associated when only household factors were included (Model 3b.3), and husband’s employment status was not associated in any models. Husband’s age changed direction between the full (Model 3b.1) and partial (3b.3) models, although it was significant in both (p < 0.05). When only community level factors were included and when all factors were included, community level percent of women with 4 or more ANC visits was associated with increased odds of delivering in a facility. However, a lower mean-stratum level acceptability of wife beating was only significantly associated with increased odds of delivering in a facility in the partial model (3b.4), but not the full model. Country of data collection was significant in all models.

**Table 7:** **Model 3b: probability of delivering at a facility: Asia**

Odds of delivering in a health facility	(1): Full	(2): Demo-graphics	(3): House-hold	(4): Community
**Woman’s Age**	1.041***	1.072***		
**Woman’s Years Education**	1.049***	1.107***		
**Total Children Ever Born**	0.789***	0.760***		
**Married**	1.110	1.017		
**Woman Being Employed**	0.966	0.759***		
**Wanted Last Pregnancy Then**	0.991	0.950		
**Year of Most Recent Birth**	1.210***	1.210***		
**Four plus ANC**	2.393***	3.402***		
**Any ANC**	1.552***	1.772***		
**Saw Doctor at ANC**	2.663***	2.942***		
**Acceptability of Wife Beating Score**	0.994	0.972		
**Urban**	1.591***		1.911***	
**Wealth Index**	1.495***		1.776***	
**Non Male Head**	1.206***		1.377***	
**Husband’s Age**	1.009**		0.989***	
**Husband’s Years Education**	1.005		1.069***	
**Husband Being Employed**	1.060		1.005	
**Percent of stratum with 4 plus ANC**	3.470***			376.0***
**Mean Stratum level Acceptability of Wife Beating Score**	1.009			0.784***
**Country**	0.940***	0.941***	0.974***	0.952***
**Constant**	0***	0***	0.160***	0.477***
**Observations**	59,081	64,261	65,650	73,003

***p < 0.01, **p < 0.05, *p < 0.1.

#### Modeling correlates of specific places of delivery and types of provider

Table 8 shows the same full model run on a variety of different more specific outcomes. Model 4 tests the correlates of delivery at a hospital, as opposed to other type of facility or home; Model 5 tests the correlates of delivering at a private facility compared to a public, other, or no facility; Model 6 tests the correlates of delivering in a public facility compared to a private, other or no facility; and Model 7 tests the correlates of delivering with a doctor compared to any other type of health professional, traditional attendant, friend, relative or no one. We highlight the variables that differed in direction or significance from the full Model of the odds of delivering in a facility (Model 1). Acceptability of wife beating was no longer associated in any of these models. In Model 4, women who delivered in a hospital were less likely to be employed, their husbands had more education, and the women were more likely to have given birth longer ago than the women who did not deliver in a hospital (husbands age was no longer significant). Additionally, higher acceptability of wife beating at the stratum level was significantly associated with lower odds of delivery at a hospital. In Model 5, younger or unemployed women, women with more children, married women, rural women, births that were longer ago, women who did not have ANC, and lower stratum-level acceptability of wife beating were more likely to deliver in a private facility. Having a non-male household head was now associated with decreased odds of delivery in a private facility, as was having a younger or a more educated husband (husband’s employment was not associated). In Model 6, a woman being employed was associated with increased odds of using a public facility; seeing a doctor for ANC was associated with decreased odds; and the husband’s education was associated with a decreased odds of using a public facility (husbands employment was not significant). Finally, in Model 7, not being employed, having the birth longer ago, not having ANC, having a younger husband, a more educated husband and a lower mean stratum acceptability of wife beating score, were associated with higher odds of delivering in a facility (urban/rural status, non-male household heath and husband employment were no longer associated significantly).

**Table 8:** **Testing associations with other types of delivery**

	Model 4: Odds of delivery at a hospital	Model 5: Odds of delivery at a private facility vs other	Model 6: Odds of delivery at a public facility vs other	Model 7: Odds of delivery with a doctor vs other
**Woman’s Age**	1.020***	0.991**	1.017***	1.037***
**Woman’s Years Education**	1.052***	1.060***	0.986***	1.064***
**Total Children Ever Born**	0.915***	1.036**	0.902***	0.854***
**Married**	0.659***	1.559***	0.711***	1.125*
**Woman Being Employed**	0.860***	0.874***	1.090**	0.699***
**Wanted Last Pregnancy Then**	0.829***	1.004	0.855***	0.951*
**Year of Most Recent Birth**	0.951***	0.897***	1.107***	0.879***
**Four plus ANC**	1.494***	1.182***	1.238***	1.573***
**Any ANC**	1.681***	0.425***	6.411***	0.479***
**Saw Doctor at ANC**	2.612***	2.277***	0.637***	10.35***
**Acceptability of Wife Beating Score**	0.994	0.989	0.990	1.008
**Urban**	1.914***	0.799**	1.408***	1.051
**Wealth Index**	1.191***	1.114***	1.253***	1.154***
**Non Male Head**	1.188***	0.891***	1.135***	0.951*
**Husband’s Age**	1.000	0.990**	1.006***	0.986***
**Husband’s Years Education**	1.031***	1.058***	0.980***	1.027***
**Husband Being Employed**	0.625**	0.667	0.869	0.899
**Percent of stratum with 4 plus ANC**	1.553*	2.606**	3.113***	1.340
**Mean Stratum level Acceptability of Wife Beating Score**	0.878***	0.790***	1.034	0.793***
**Country**	0.986**	0.970***	0.986**	0.978***
**Constant**	8.8e + 42***	3.3e + 94***	0***	1.1e + 111***
**Observations**	213,255	114,334	213,255	213,204

***p < 0.01, **p < 0.05, *p < 0.1.

## Discussion

Our results confirm many of the findings from the systematic review by Moyer and Mustafa (2013), but are in conflict with other findings [3]. Our findings support existing literature that find maternal education, parity, urban residence, higher wealth quintile, any/4 or more ANC visits, seeing a doctors for ANC, living in a household with a non-male head, higher husband’s education and the community level percent of women who had 4 or more ANC visits were associated with higher odds of a facility delivery. Similar to their findings, we find mixed results for the significance of marriage on facility delivery. If the measure of wife beating is assumed to be a measure of women’s autonomy, we find some evidence that more empowered women were more likely to deliver in a facility, as would be expected from the previous literature. Additionally, we find some evidence that the community score on acceptability of wife beating is associated with facility delivery, although this is not consistent across models.

Past literature suggests that younger maternal age is associated with increased odds of delivering in a facility. Literature in Asia also paint a mixed picture, with some studies predicting that higher maternal age was associated with delivering in a facility [8, 32–34], while other studies found a negative association [7], and others found no association [4, 9–11, 16, 21, 22, 35]. All of our models find that older maternal age is significantly associated with the odds of facility delivery in Africa and Asia.

We find that, where significant, maternal employment was negatively associated with the odds of facility delivery, which is the opposite of what Moyer and Mustafa (2013) found [3]. Additionally, we find that desired pregnancies were less likely to have been delivered in a facility, again, contrary to their findings. Contrary to past findings, we find that in some models husband’s employment status was negatively associated with the odds of delivering in a facility. This could be due to the fact that we use a binary variable of not employed/any employment due to country-level coding differences, and past literature had focused on farmers compared with non-farmers.

When modeled separately there are few differences in correlates of delivering with a provider and delivering at a facility between Africa and Asia. Interestingly, year of most recent birth is not significant in Africa, although it is in Asia and in the full models. This suggests that perhaps time trends are more important in Asia than in Africa, which supports the trends seen in the graphs. Living in a household with a non-male head is not associated in the full model in Africa, although it is in all other models. Perhaps male permission or the role of male decision making is less important in Africa compared to Asia. In Asia, being married, whether the pregnancy was desired, and the acceptability of wife beating score are not associated with facility delivery. Since these factors are associated in the full country model, it is clear that this is dominated by the African experience.

When we look at more specific outcomes (delivering in a private health facility, public health facility, hospital or with a doctor), a few small differences emerged. Particularly, women who delivered in a private facility seemed to stand out: they are more likely to be younger, married, have more children, have not had ANC and are rural.

Across the board, having any ANC (and 4 or more ANC visits) was consistently, positively, and strongly (high odds ratios) correlated with facility delivery. The percent of women in a stratum that had ANC was also very strongly correlated with facility delivery in all of the models. With such a large sample size, odds ratios that are just above or below 1, yet statistically significant, might not be that meaningful. That those variables related to ANC use at both an individual and community level have odds ratios over 2 consistently across models therefore warrants further attention. Past literature has noted that the same type of women who deliver in a facility will seek ANC, therefore attributing causation from ANC to facility delivery is not appropriate. However, even after controlling for other individual, household and community level factors, we see such a strong correlation between ANC and facility delivery, that this relationship appears to be the strongest of those explored in this analysis.

### Limitations

Cross-sectional data, such as the DHS surveys, preclude the ability to draw causal conclusions, limiting our analysis to look at correlates, rather than predictors or determinants of facility delivery. In order to analyze the current situation for facility delivery, we limited the sample to surveys collected since 2003 (10 years before the time of this analysis). Within that, we limit the sample to births since 1993. However, some of the data is more recent than others and therefore we are seeing a relatively wide window on the current status of rates and trends in facility delivery. Ideally, we would have data from all countries in the same year so as to make a better comparison.

Another set of limitations revolves around the fact that this is individual, self-reported, and for some indicators relating to past births, retrospective data. This type of survey data can therefore suffer from recall bias, or other errors due to lack of knowledge on the part of the respondent (for example, classifying anyone in a health facility as a “doctor” without knowing their actual credential). These biases are unavoidable in these types of surveys.

Many of the variables that past literature found to be associated with facility delivery are not in, or could not be modeled within the DHS. Particularly relevant is that there were only a limited number of community level and facility related factors that we could include. Additionally, we were unable to include any of the macro-level factors that other scholars have found to be associated with facility delivery. There are a number of indicators that are only in some DHS’s (such as health insurance or knowledge of pregnancy risk factors), and we therefore had to exclude these issues because they would have dramatically reduced the sample size.

We were also limited because there may be other factors that are related to a woman’s odds of delivering in a facility that are not in the DHS, especially those relating to maternal complications, quality of care and perceptions of quality, and access to facilities (distance, cost, etc.). It is possible that some of the associations that we have identified are actually related to other factors such as pregnancy complications, distance to facility, etc., rather than to facility delivery itself.

## Conclusions

This study is a multi-country analysis of the drivers of facility deliveries across regions.. It examines the individual, family, community, and facility level factors that lead to facility deliveries. The study finds that the majority of women surveyed in the 43 DHSs explored in this study are delivering in some type of facility with a health care provider. Rates are lower in Asia than in Africa, and private facilities make up a larger proportion of delivery facilities in Asia than in Africa. Disparities exist between rich and poor, and urban and rural populations. There is still a need to increase accessibility to facilities for delivery for poorer and more rural women in all regions of the globe.

A vast number of previous studies have looked at drivers of facility deliveries in certain regions or countries, often using small datasets. This analysis combines information about potential drivers of facility delivery from these studies into one multi-level analysis of countries in Africa and Asia. We find support for the majority of previous findings on a multi-country level, and few differences between African and Asian regions. Demographic, household and community level factors all contribute to a woman’s odds of delivering in a facility. The most powerful correlation in this analysis was between a woman having ANC or a larger percent of women in a community having ANC, and facility delivery. Even after controlling for other variables, this was the strongest and most consistent relationship. Understanding how ANC and facility delivery are linked is key. Do the same type of women who go to ANC go to a facility to deliver? Or does the very act of getting women into ANC set her on a “facility” track? Or is there something that happens in ANC that convinces women to deliver in a facility? Further longitudinal study is needed to explore this.

More research is also needed to understand other drivers of facility delivery, so as to be better able to target women who do not currently deliver in facilities so they can have access to the care they need at the time of delivery. As discussed above, some of the drivers may be related to maternal complications which would make it more likely for a woman to deliver at a facility. Other factors such as cost, distance, transportation, and policy-related factors like bans on home delivery and cash incentive programs also might be influencing decision making. We were unable to include factors such as these in our analysis. More research is needed to pinpoint which other factors are the most important “missing” drivers in women’s decision-making, so we can understand what barriers remain and what incentivizes women to deliver in facilities. It is time to move beyond individual, demographic factors and focus on the community, facility and policy levels. These findings clearly show that more and more women are delivering in facilities in Africa and Asia, suggesting that perhaps policy makers should begin to focus more on the quality of care that women receive in facilities and ensuring that facilities are not overburdened.
